# Neoadjuvant Chemotherapy and Whole Ventricular Irradiation for Pure Intracranial Germinoma: A Comparison of Three Brain-Sparing Techniques

**DOI:** 10.7759/cureus.13670

**Published:** 2021-03-03

**Authors:** Jason Nosrati, Arthur J Olch, Ryan J Abel, Kenneth Wong

**Affiliations:** 1 Department of Radiation Medicine, Northwell Health Cancer Institute, Lake Success, USA; 2 Department of Radiation Oncology, Keck School of Medicine of the University of Southern California, Los Angeles, USA; 3 Radiation Oncology Program, Children's Hospital Los Angeles, Los Angeles, USA; 4 Radiation Oncology, Mitchell Memorial Cancer Center, Owensboro, USA

**Keywords:** germinoma, radiotherapy, pediatric germinoma, intracranial germinoma, whole ventricular irradiation, pediatric brain tumor

## Abstract

Objective

To quantitate and compare the dosimetric properties of three brain-sparing radiation therapy techniques for pure intracranial germinomas with dose-volume analysis of target and normal brain structures.

Methods

We identified 18 patients with central nervous system (CNS) germinoma who had achieved local control and had excellent neurocognitive outcomes. Four patients who were treated with a simultaneous integrated boost (SIB) plan of 22.5Gy to whole ventricle (WV) and 30Gy to primary were re-planned with 24Gy to WV-only and the Children’s Oncology Group (COG) protocol of 18Gy to WV with a sequential boost to 30Gy. Organ-at-risk (OAR) doses for hippocampi, temporal lobes, whole brain, whole brain minus whole ventricles planning target volume (WB-WVPTV), WVPTV, and boost volume were comparatively studied.

Results

For patients treated with the SIB plan, an excellent neurocognitive function has previously been shown to be well preserved. Three-year event-free survival (EFS) and overall survival (OS) for this group have also previously been demonstrated (89.5% and 100%, respectively). Mean and integral OAR doses were comparable between SIB and WV-only plans but were lower for COG plans. Whole brain, whole brain minus WVPTV, and temporal lobe V20, V18, and V12, as well as hippocampi V20, V25, and V30, were comparable between SIB and WV-only plans but were lower for the COG plans.

Conclusion

Compared to the WV-only method, the SIB plan permits more dose to the primary site by 6 Gy without compromising neurocognitive control. While maintaining the 30Gy boost, the COG protocol reduces the WVPTV dose to 18Gy. It remains to be seen whether WV dose reduction risks reducing local control.

## Introduction

Intracranial germ cell tumors represent 3%-11% of pediatric central nervous system (CNS) malignancies, of which two-thirds are pure germinomas and one-third are non-germinomatous germ cell tumors (NGGCT) and mature teratomas [1-5]. Pure germinomas most often occur in the suprasellar cistern (49%) and pineal gland (37%), with 8% presenting as bifocal, in both regions. Three-quarters of diagnosed females present with suprasellar primary sites, whereas two-thirds of males present with pineal primary sites [6].

Due to the highly vascular nature of these tumors and with good response to chemotherapy, complete resection only plays a minor role in managing these tumors [4]. The previous standard of care was 30 or 36Gy craniospinal irradiation (CSI) with a respective 14 or 15Gy boost to the primary site [4,7]. CSI has cure rates of greater than 90% [8], but the delivery of high CSI doses to the entire craniospinal axis is associated with significant late effects such as impaired bone growth, neurocognitive deficits, and endocrine toxicities [2]. Knowledge of these toxicities has led to a re-evaluation of treatment and a shift in the focus of research from increasing cure rates to maintaining cure rates by using chemotherapy followed by radiation therapy (RT) with lower doses [2-3]. This reduced dosing may result in decreased incidence and severity of late effects, including second malignancies and neurocognitive deficits [5,9].

The current standard of treatment for these tumors in most pediatric patients involves sequential chemotherapy and reduced dose whole ventricular irradiation followed by primary site boost [3]. Some Japanese groups choose to forgo the boost and treat the whole ventricles only to a somewhat greater dose [10-11]. Alternatively, a simultaneous integrated boost (SIB) technique can be used to deliver the boost concomitantly [5]. Our standard had been to treat patients at our institution with 22.5Gy ventricular field irradiation with SIB to the primary site to a total dose of 30Gy over a total of 15 fractions.

This continued until 2012 when we began enrolling patients in the current Children’s Oncology Group (COG) ACNS1123 phase 2 clinical trial. This trial consists of a neoadjuvant chemotherapy regimen of carboplatin and etoposide followed by reduced-dose whole ventricular intensity-modulated radiation therapy (IMRT) to 18 Gy with a local sequential boost to the tumor to a total of 30Gy in 20 fractions [12].

The purpose of this study is to quantitate and compare dosimetric properties of three brain-sparing treatment techniques for pure intracranial germinomas with a dose-volume analysis of normal brain structures: the SIB technique [5,13], a Japanese 24Gy WV-only technique [10-11], and the COG technique [12].

The preliminary data of this study were presented at the Radiological Society of North America (RSNA) 2018.

## Materials and methods

**Figure 1:**
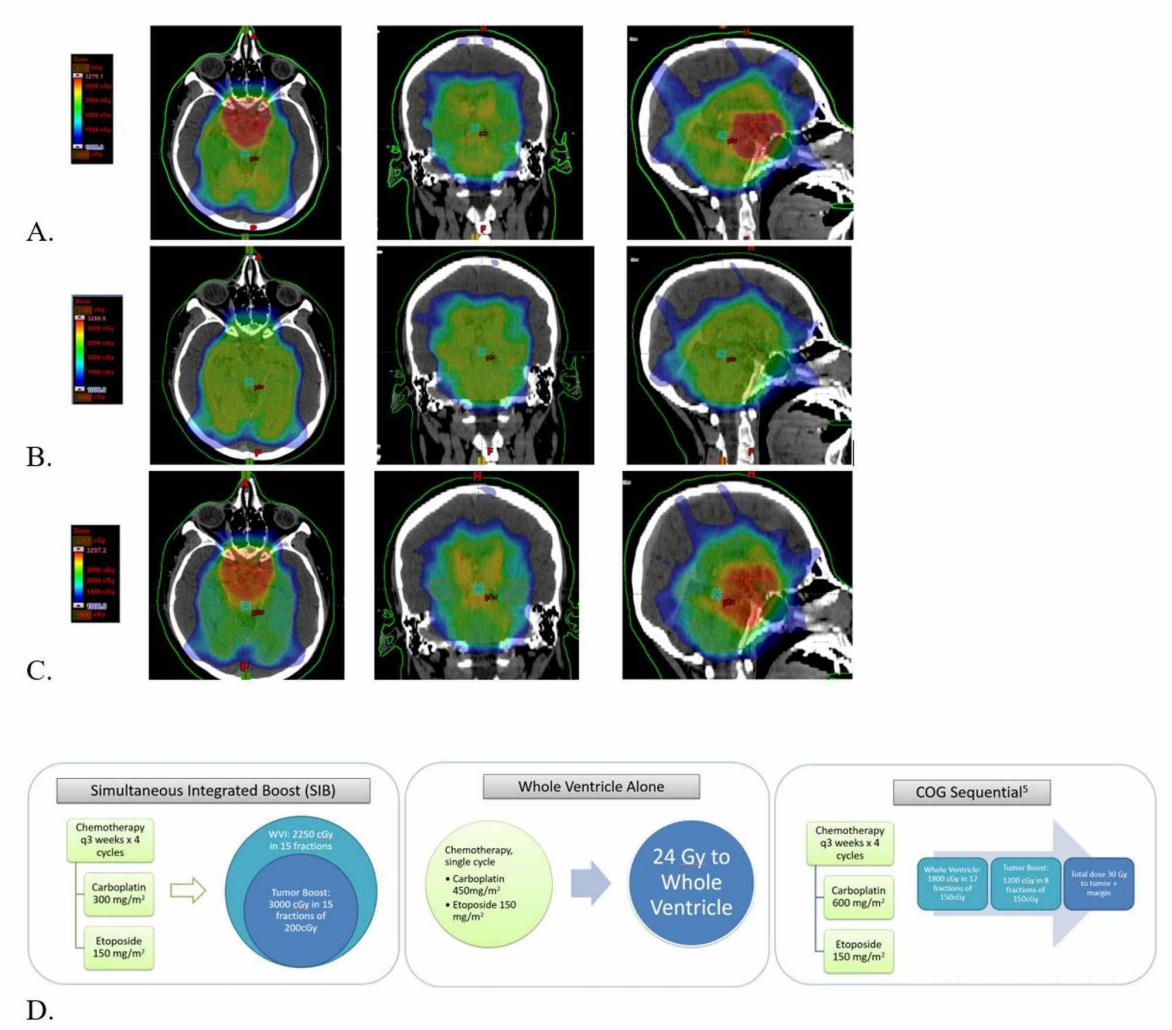
Dose color washes for the axial, coronal, and sagittal plane for one example case of each treatment method and outlines for each paradigm A. SIB (Top, 10.0-32.7Gy), B. WVI (Middle, 10.0-32.8Gy) C. COG (Bottom, 10.0-33.0Gy), D. Treatment paradigm for each method. SIB: simultaneous integrated boost; WVI: whole ventricular irradiation; COG: Children's Oncology Group

Figure 1 shows the three brain-sparing treatment techniques for pure intracranial germinomas with dose-volume analysis of normal brain structures.

Patient population

**Figure 2:**
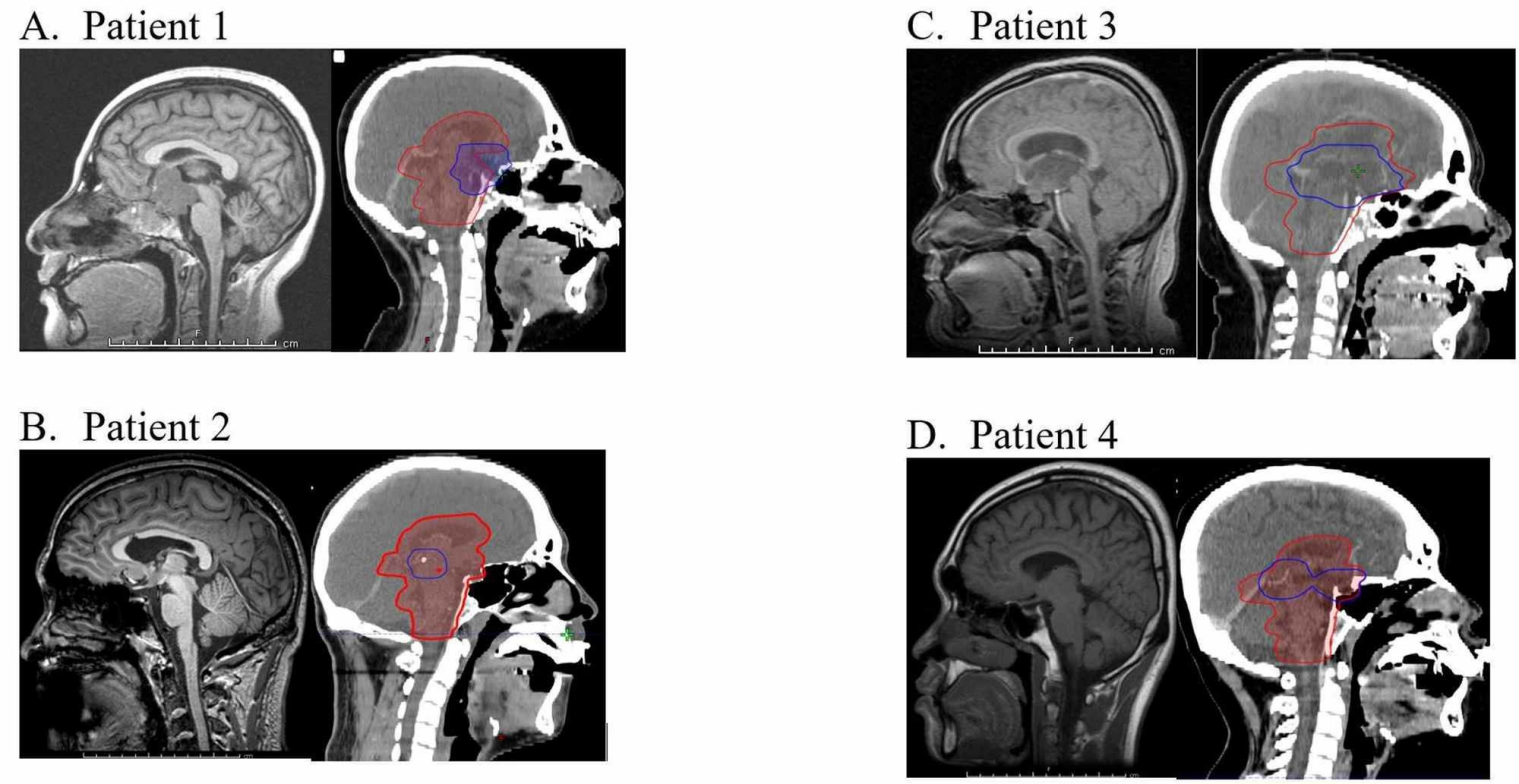
Tumor locations for four patients A-D. Sagittal MRI (left) and Eclipse TPS images with target contours – blue for PTV; red for WVV-PTV (right) TPS: treatment planning system; PTV: planning target volume; WVV: whole ventricular volume Eclipse: Varian Medical Systems, Palo Alto, CA

After approval from the IRB, we identified a cohort of 18 CNS germinoma patients diagnosed at our institution between 2003 and 2009 and treated with a simultaneous boost paradigm. From this cohort, four patients representing a large range of primary site volumes located in the suprasellar and/or pineal regions were examined retrospectively. Excellent neurocognitive and local control outcomes for all patients in the present study were previously reported by our group [5]. In the present study, we further analyze these patients in terms of OAR dose-volume metrics and dosimetrically compare the simultaneous boost paradigm to two other paradigms used across the world today. Computed tomography (CT) images of our four patients showing WV and tumor size are shown in Figure 2. Corresponding WV and tumor volumes are shown in Table 1. Informed consent was waived by the IRB due to the retrospective nature of our study.

**Table 1:** Beam angles for the 10-beam non-coplanar plan used for all plans in this study Angles are in the "Varian standard" rather than the IEC coordinate system. Varian: Varian Medical Systems, Palo Alto, CA; IEC: International Electrochemical Commission

Beam name	Gantry (deg)	Collimator (deg)	Couch (deg)
POSTSAG	305	0	90
ANTSAG	220	0	90
RASO	240	0	140
RPSO	325	0	148
LPIO	75	0	170
LAIO	135	0	170
LASO	120	0	220
LPSO	35	0	212
RPIO	285	0	190
RAIO	225	0	190

Comparison of OAR dose-volume metrics

The hippocampi, temporal lobes, whole brain, whole ventricular volume (WVV), and boost volume were manually contoured for each patient, and whole brain minus whole ventricular PTV (WB-WVPTV) was calculated from these contours. The RTOG 0933 Hippocampal Contouring Atlas [14] and an internal atlas at our institution (Velocity, Varian Medical Systems, Palo Alto, CA) were used to guide hippocampal contouring and contouring of all other structures, respectively. SIB plans that were initially used to treat these patients were re-planned with the Japanese and current COG treatment paradigms to make a total of 12 plans. Eclipse (Version 13, Varian Medical Systems, Palo Alto, CA) was used to plan step-and-shoot IMRT treatments using 6 MV photons. All 12 plans used the same 10 non-coplanar beam arrangement (Table 1). Each plan was optimized with the same PTV dose coverage of at least 98% of the PTV receiving at least 98% of the prescribed dose.

**Figure 3:**
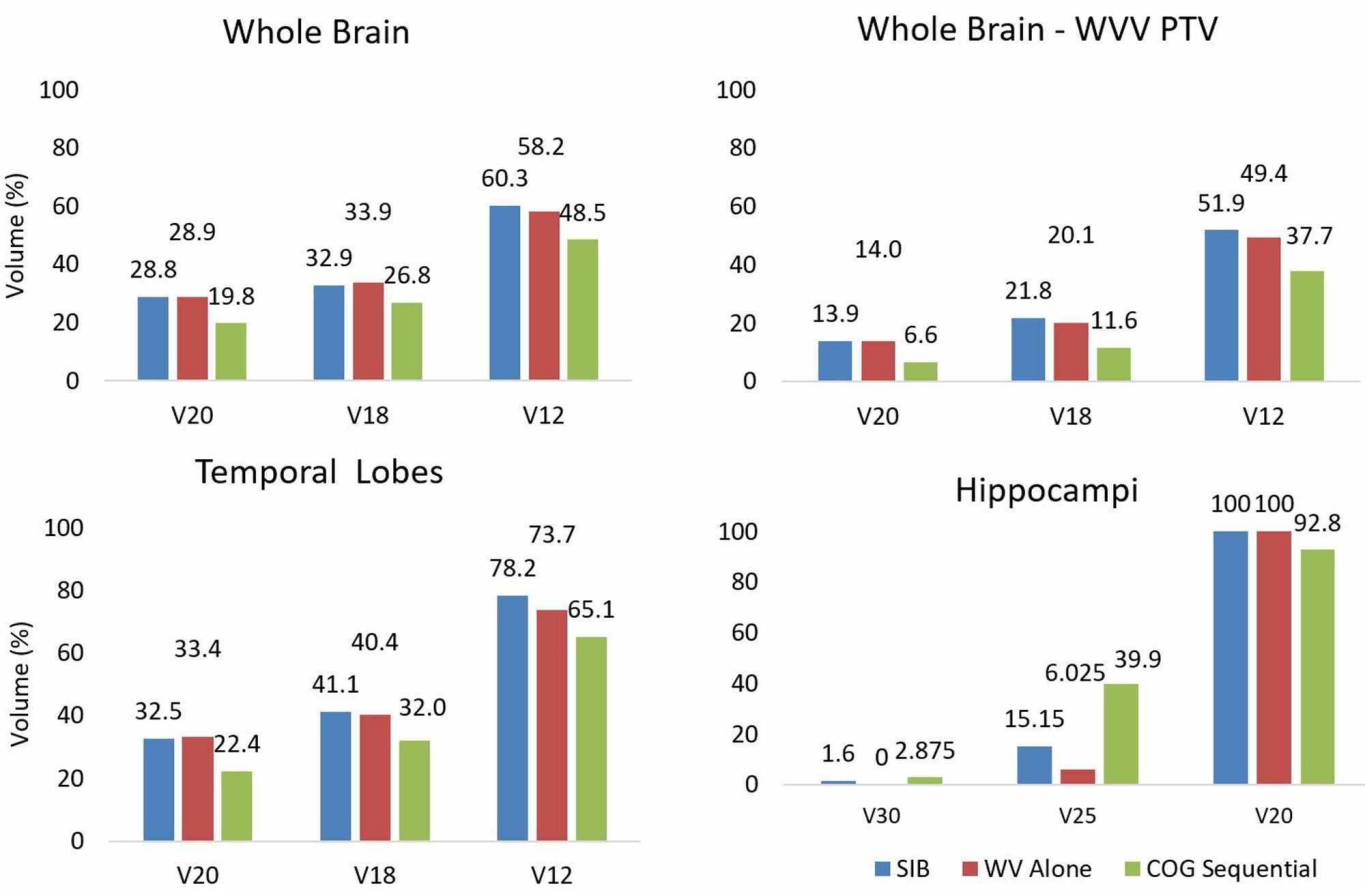
Comparison of the three treatment paradigm’s mean dose-volume metrics for the four patients Mean V20Gy, V18Gy, and V12Gy dose metrics shown for whole brain, whole brain minus PTV, temporal lobe, and mean V30Gy, V25Gy, and V20Gy for hippocampi.

Volume and averaged mean, minimum, maximum, and integral dose for targets and critical structures across four patients are shown in Table 2. The integral dose was calculated as the area under the dose-volume histogram (DVH) curve for a particular structure of interest, which is approximately the same as mean dose x volume and is reported in Joules [15]. The absolute dose to 95% and 5% of the PTV volume (D95 and D5, respectively) were recorded for each plan, and averages across four patients were noted in Table 3. The percentage volume receiving at least 12, 18, or 20Gy (V12, V18, V20) were recorded for the whole brain, temporal lobes, and WB-WVPTV. V20, V25, and V30 were recorded for hippocampi. Averages across the four patients for these measurements are reported in Figure 3.

**Table 2:** Tumor site, volume, and WVV recorded from the Eclipse TPS for all patients TPS: treatment planning system; WVV: whole ventricular volume Eclipse: Varian Medical Systems, Palo Alto, CA

Patient #	Tumor Site	Tumor Volume (ml)	WVV (ml)
1	Small Suprasellar	36.6	215.8
2	Pineal	12.7	279.3
3	Large Suprasellar	61.0	306.7
4	Bifocal	33.0	211.3

**Table 3:** Dose-volume histogram average mean dose, min dose, max dose, volume, and integral dose for PTVs and critical OARs WV: whole ventricle; PTV: planning target volume; OAR: organ at risk

Organ	Mean dose (cGy) (min,max) (SIB)	Mean dose (cGy) (min,max) (WV alone)	Mean dose (cGy) (min,max) (COG)	Volume (ml)	Integral dose (J) (SIB)	Integral dose (J) (WV alone)	Integral Dose (J) (COG)
Whole Ventricular Volume	2486.4 (1788.2, 3154.3)	2439.9 (1888.8, 2582.8)	2396.0 (1445.3, 3168.0)	253.3	6.29	6.17	6.08
Boost Volume	3052.6 (2805.0, 3154.1)	2434.1 (2199.8, 2530.6)	3065.1 (2788.1, 3168.1)	35.8	1.10	0.87	1.10
Hippocampi	2384.0 (2174.4, 2961.5)	2424.4 (2285.1, 2502.1)	2375.2 (1914.0, 3057.6)	2.6	0.06	0.06	0.06
Temporal Lobes	1632.6 (502.2, 3058.8)	1622.1 (461.8, 2554.2)	1444.7 (423.0, 3099.5)	88.1	1.45	1.44	1.28
Whole Brain	1500.9 (104.6, 3152.0)	1470.8 (104.6, 2582.8)	1341.3 (88.5, 3168.5)	1408.0	21.11	20.67	18.84
Brain-WV PTV	1292.3 (105.6, 3112.0)	1462.2 (712.5, 2478.3)	1050.3 (167.0, 2808.5)	1948.1	15.93	14.43	10.15

## Results

For all plans, target volume coverage was comparable. The D98 was at least 98% of the prescription dose for all plans. The ratio of WV to total brain volume ranged from 13.8% to 21.8%. Mean and integral OAR doses were comparable between the SIB and WV alone plans but were generally lower for the COG plan (Table 2). WV and boost D5, as well as boost D95, were similar for the SIB and COG plans but lower for the WV-alone plan (Table 3). D95 for all OARs are similar between the SIB and WV-alone plans but lower in the COG plan. D5 for all OARs are similar between the SIB and COG plans but lower in the WV alone plan. Figure 3 and Table 4 show that whole brain, whole brain - WVV PTV and temporal lobe V20, V18, and V12 as well as hippocampi V20, V25, and V30 were comparable between the SIB and WV-alone plans but were, in most cases, greater than the COG plans.

**Table 4:** Dose-volume comparison for critical structures A. D95 and D5 PTV and OAR doses (cGy), B. WVV-PTV and boost V100 doses as % of the prescribed dose (cGy) WV: whole ventricle; WVV: whole ventricular volume, PTV: planning target volume; OAR: organ at risk

Organ	D95 (SIB)	D95 (WV alone)	D95 (COG)	D5 (SIB)	D5 (WV alone)	D5 (COG)
Whole Ventricles	2242.0	2380.1	1914.6	3011.0	2491.1	3065.5
Boost	2978.5	2394.3	3002.4	3100.2	2472.5	3114.9
Hippocampi	2248.1	2373.6	2081.7	2562.0	2469.1	2560.0
Temporal Lobes	905.5	860.5	756.2	2470.2	2144.0	2443.0
Whole Brain	484.1	474.0	409.4	2604.2	2460.2	2684.5
Brain-WV PTV	449.5	443.6	409.2	2229.8	2149.6	1915.5

Organ	V100 (SIB)	V100 (WV alone)	V100 (COG)
Whole Ventricular Volume	92.8	86	88.7
Boost volume	100.0	88.1	92.2

## Discussion

We conducted this analysis across four patients with varying WV and tumor volumes. These patients were all treated at our institution with 22.5Gy with SIB to 30Gy [5]. Excellent neurocognitive function was previously reported for this cohort at three years [5]. This included verbal and nonverbal intellectual functioning, working memory, processing speed, executive functioning measures of complex attention skills, involving response inhibition, cognitive ﬂexibility/set-shifting, and planning, immediate, and delayed verbal and nonverbal memory, depression, anxiety, and social functioning.

Likewise, event-free survival (EFS) and overall survival (OS) for this group were previously shown to be 89.5% and 100%, respectively, at three years [5]. Similarly, 10-year EFS and OS for the Japanese plan giving 24Gy to the WV without boost were previously shown to be 89% and 100%, respectively [10-11]. Compared to the Japanese 24Gy WV-only plan, the SIB method (22.5Gy to WVV with primary boost to 30Gy) reduced the dose to the WVV by 1.5Gy while maintaining excellent survival and neurocognitive outcomes [4-5,10-11]. The COG protocol (18Gy to WVV with primary boost to 30Gy) further reduces the dose to normal brain by reducing the WVPTV dose to 18Gy while maintaining the 30Gy boost PTV dose. It remains to be seen whether this risks lack of control for the WVPTV compared to the SIB or Japanese treatment.

One notable study compared three dose-sparing techniques in a similar manner [16]. Patients in their study were not limited to germinoma but rather comprised five germinoma patients and six NGGCT patients. Their patients were initially treated with SIB (32.4-26 Gy to the WVV and 54 Gy to the primary tumor) and replanned with whole ventricular irradiation (WVI) with opposed-lateral beams plus IMRT boost to the primary tumor as well as IMRT to the WVV with sequential IMRT boost to the primary tumor. The results of this study showed better dose-sparing of OARs with SIB as compared to both sequential IMRT and opposed-laterals plus IMRT. For example, there was a 10%-13% and 7%-11% decrease in mean dose to the whole brain, 17%-21% and 33%-36% decrease in mean dose to temporal lobes, 7%-30% and 28%-40% decrease in mean dose to the cochlea, and 16%-25% and 15%-28% decrease in the mean dose to optic nerves compared to sequential IMRT and opposed-laterals plus IMRT, respectively. Considering our results in conjunction with the results of their study, it could be reasonable to consider the replacement of the sequential boost with a SIB boost in future clinical trials.

One notable limitation of our study is that we have only included patients with a complete response to chemotherapy. The COG trial suggests that for patients without complete response, one should deliver 24Gy to the WVV with a 12Gy sequential boost [12]. It may be reasonable to conduct a separate study to consider the role of SIB in patients without complete chemotherapy response.

## Conclusions

The SIB technique offers excellent tumor control (with five fewer fractions than the COG method) without sacrificing neurocognitive function. Although the results of the COG ACNS1123 trial are not yet available, this modality treats the WVV to a lower overall dose, potentially risking ventricular recurrences. Therefore, escalating the dose beyond that used in the COG ACNS1123 trial and using SIB treatment could potentially improve local control without sacrificing long-term patient outcomes. Although the Japanese method may appear to be more sparing of normal brain due to the lack of a boost phase, in fact, about the same volume of normal brain receives potentially harmful doses as the SIB technique. We hope that this work will encourage further consideration of the simultaneous boost technique in the treatment of pure CNS germinoma.
